# Identification of a Candidate Rad1 Subunit for the Kinetoplastid 9-1-1 (Rad9-Hus1-Rad1) Complex

**DOI:** 10.3390/biology3040922

**Published:** 2014-12-19

**Authors:** Stuart A. MacNeill

**Affiliations:** School of Biology, University of St Andrews, North Haugh, St Andrews, Fife KY16 9ST, UK; E-Mail: stuart.macneill@st-and.ac.uk; Tel.: +44-1334-467268

**Keywords:** 9-1-1 complex, sliding clamp, kinetoplastid

## Abstract

The trimeric 9-1-1 (Rad9-Hus1-Rad1) complex plays an important role in the eukaryotic DNA damage response by recruiting DNA repair factors and checkpoint mediators to damaged sites. Extensively characterised in mammals and yeast, evidence is now emerging that 9-1-1 function is conserved beyond the relatively narrow evolutionary range of the Opisthokonts. Kinetoplastid Rad9 and Hus1 proteins have been identified and shown to be involved in the DNA damage response but Rad1 has remained elusive. In this study, PSI-BLAST iterative database searching, phylogenetic and structural modeling techniques are used to identify and characterise candidate Rad1 proteins in kinetoplastid organisms.

## 1. Introduction

Sliding clamps play vital roles in DNA metabolism in all forms of cellular life [1]. In eukaryotes, two distinct sliding clamp complexes have been identified: PCNA (proliferating cell nuclear antigen) and the 9-1-1 (Rad9-Hus1-Rad1) complex. PCNA is a ring-shaped homotrimer that is crucial for many aspects of DNA replication and repair. The PCNA ring is loaded onto DNA in an ATP-dependent manner by a clamp loader complex (replication factor C, RFC) and acts both as a processivity factor for the replicative DNA polymerases (by tethering these enzymes to their DNA substrates) and as a landing platform for numerous enzymes involved in Okazaki fragment maturation and various DNA repair pathways [1]. The 9-1-1 complex also functions in DNA damage repair but has an additional role in activating the DNA damage checkpoint to ensure that cell cycle progression is halted until repair is complete [2]. This complex also forms a trimeric ring but unlike PCNA, the ring is composed of three different subunits: Rad9, Hus1 and Rad1. Each of these proteins is distantly related to PCNA at both sequence and structure levels, indicative of their shared ancestry. Like PCNA, loading of 9-1-1 onto DNA requires the action of a clamp loader complex, the Rad17-RFC complex [3]. Loading occurs at damaged sites and leads to the recruitment of additional repair factors including, in human cells, the DNA glycosylases NEIL1 and TDG, the APE1 phosphodiesterase, DNA polymerase β and DNA ligase I [2].

Kinetoplastids are a group of unicellular flagellated protozoa that includes organisms responsible for several neglected tropical diseases, including African sleeping sickness (trypanosomiasis, caused by *Trypanosoma brucei*), Chagas disease (*T. cruzi*) and various types of leishmaniasis (diverse *Leishmania* species). Despite their impact on human health, many aspects of kinetoplastid biology remain understudied, including mechanisms for maintaining genome integrity in the face of endogenous and exogenous DNA damage. Understanding how kinetoplastid organisms respond to damage may highlight vulnerabilites that can be exploited as therapeutic targets.

The extent to which the function of the 9-1-1 complex is conserved across eukaryotic evolution remains unclear, as almost everything that is known about this factor comes from studies on Opisthokont organisms, in particular yeasts (*Saccharomyces cerevisiae* and *Schizosaccharomyces pombe)* and mammals [2]. In two recent studies on *Leishmania major*, Tosi and co-workers identified and characterised the first kinetoplastid homologues of Hus1 and Rad9, encoded by the *L. major* genes LmjF.23.0290 and LmjF.15.0980 respectively, but were unable to identify homologues of Rad1 [4,5]. Data from these studies is consistent with the Hus1 and Rad9 proteins functioning in the DNA damage response. Heterozygous LmHus1^+/−^ knock-out strains, in which endogeneous Hus1 levels are reduced to ~50% of wild-type, display impaired growth, premature entry into stationary phase, telomere shortening and a failure to arrest cell cycle progression when exposed to the DNA damaging agents hydroxyurea (HU), methylmethanesulphonate (MMS) and camptothecin (CPT), while homozygous LmHus1^−/−^ knock-outs appear to be inviable [4]. Overexpression of LmHus1 enhances the cell’s capacity to deal with replication stress: LmHus1 overexpressing cells display increased resistance to both HU and MMS, improved recovery from HU arrest and enhanced DNA repair [5]. In contrast, LmRad9 overpression sensitizes cells to MMS and does not lead to HU or CPT resistance [4]. LmHus1 and LmRad9 can be co-immunoprecipitated from *L. major* extracts, consistent with them forming part of a 9-1-1 complex.

Overall, these results point to the identified Hus1 and Rad9 proteins playing similar roles to their opisthokont counterparts [4,5] but leave many questions to be answered about how these roles are enacted. In particular, the lack of candidate kinetoplastid Rad1 proteins potentially calls into question the existence of the 9-1-1 complex in these organisms. In this regard it is noteworthy that certain eukaryotic viruses encode PCNA orthologues that form either monomeric [6] or dimeric [7] sliding clamps and that trimerisation is therefore not a prequisite for function. In this paper, I report the identification of a candidate kinetoplastid Rad1 protein.

## 2. Experimental Section

PSI-BLAST searches were performed against the NCBI Reference Sequence (RefSeq) database using default PSI-BLAST parameters [8,9]. Modelling was performed using the Phyre^2^ modelling server [10,11]. Protein structures were visualised using MacPyMOL (http://www.pymol.org).

## 3. Results and Discussion

In order to identify candidate Rad1 proteins in kinetoplastids, iterative PSI-BLAST searches [8,9] were performed, initially using the well-characterised 282 amino acid human Rad1 protein as the query sequence (UniProtKB accession number O60671). After three iterations several kinetoplastid proteins were detected with E-values ranging from 2e-12 to 3e-06. By way of comparison, several archaeal PCNA proteins (both homo- and heterotrimeric types) were detected with similar E-values at this iteration. The kinetoplastid proteins (see Table S1 for accession numbers) ranged in length from 301–362 amino acids and shared around 15%–20% sequence identity with human Rad1. In addition to using PSI-BLAST, *L. major* Rad1 was also detected (with 100% confidence) using the BackPhyre facility in Phyre^2^ [10,11] with human Rad1 structure (PDB:3G65, chain B) as the query sequence.

**Figure 1:**
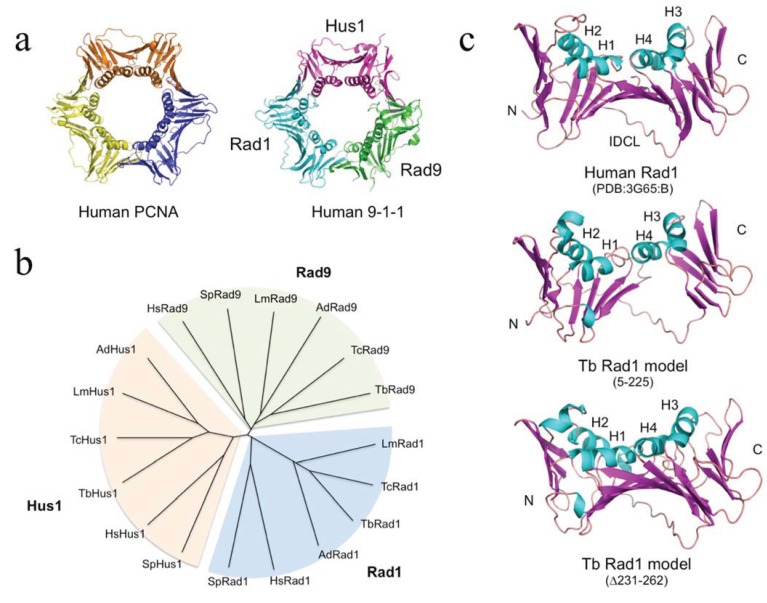
The kinetoplastid 9-1-1 complex. (**a**) Human sliding clamp structures: proliferating cell nuclear antigen (PCNA) (PDB:1VYM) and 9-1-1 (PDB:3G65). Individual subunits of the 9-1-1 are labelled; (**b**) Phylogenetic analysis of human, fission yeast and kinetoplastid 9-1-1 complex proteins. Abbreviations: Hs, *Homo sapiens*; Sp, *Schizosaccharomyces pombe*; Tb, *Trypanosoma brucei*; Tc, *T. cruzi*; Lm, *Leishmania major*; Ad, *Angomonas deanei*. UniProtKB accession numbers are given in Supplementary Table S1; (**c**) Structural model for residues 5–225 of the putative *T. brucei* Rad1 protein generated by Phyre^2^ [10,11] alongside the human Rad1 structure (chain B from PDB:3G65). Abbreviations: H1-H4, α-helices 1–4; IDCL, interdomain connector loop. Protein structures were visualised using MacPyMOL.

At the TriTryp database [12], the kinetoplastid Rad1 proteins are annotated as conserved hypotheticals. Phylogenetic analysis carried out using Clustal X [13] groups the newly identified kinetoplastid Rad1 proteins from *T. brucei*, *T. cruzi*, *L. major* and the insect trypanosomatid *Angomonas deanei* with human and fission yeast Rad1 in a cluster distinct from the Rad9 and Hus1 clusters (Figure 1B). As previously observed in Opisthokont species [14], the kinetoplastid Rad1, Rad9 and Hus1 proteins are more closely related to each other than to the PCNA proteins [15]. PCNA has also diverged less over evolution than the Rad1, Rad9 and Hus1 proteins: pairwise amino acid sequence comparison of PCNA, Rad1, Rad9 and Hus1 proteins from *T. brucei* and *T. cruzi*, for example, shows these to be 78%, 55%, 33% and 40% identical, respectively. This likely reflects the central role played by PCNA in many DNA transactions constraining changes in protein sequence.

In order to probe the likely extent of structural similarity between the newly identified proteins and well-characterised Rad1 proteins [14,16], the sequence of the putative *T. brucei* Rad1 protein was submitted to the Phyre^2^ modelling server [10,11]. 74% of the TbRad1 (amino acids 5–225) sequence was modelled with >90% confidence using the full-length human Rad1 protein (PDB:3G65, chain B) as a template. Individual sliding clamp proteins are broadly similar in structure: in each subunit a series of β-strands form the outside of the ring-shaped clamp structure, while the central cavity of the ring is lined by four α-helices (Figure 1A). The TbRad1 model retains the four α-helices (H1-H4), much of the supporting β-strand network and the interdomain connector loop (Figure 1C, middle part). Absent from the model are the three β-strands that follow α-helix H4 in human Rad1. This short region (corresponding to residues 237–271 in human Rad1) is reasonably well-conserved in the kinetoplastid Rad1 proteins but is separated from the H4 helix by a poorly conserved kinetoplastid-specific insertion (often of low sequence complexity) that ranges in length from 30–50 amino acids depending on species and which disrupts model building: resubmission of the TbRad1 sequence to Phyre^2^ with the insertion sequence (amino acids 231–262) deleted produces a model in which 95% of the sequence is modelled with 100% confidence and where the C-terminal β-strand are correctly modelled (Figure 1C, lower part). Interestingly, it has been argued previously that the side-chains of two residues within this *C*-terminal region (Phe250 and Cys272) prevent human Rad1 from binding to proteins with PIP (PCNA interacting protein) motifs [17], by occupying space that could otherwise be occupied by the central hydrophobic residue in the PIP sequence [14]. In the kinetoplastid proteins Phe250 is replaced by glutamine and Cys272 by either serine (*T. brucei*, *T. cruzi*), methionine (*L. major*) or leucine (*A. deanei*), suggesting that these proteins are also unable to bind PIP motif proteins. The identification of a Rad1 protein will permit biochemical investigation of the kinetoplastid 9-1-1 complex and allow such questions to be answered.

## 4. Conclusions

The identification of a candidate Rad1 protein completes the complement of kinetoplastid 9-1-1 complex subunits and paves the way for detailed genetic, biochemical and structural analysis of the role of the 9-1-1 complex in DNA response pathways in these early-branching eukaryotes.

## Supplementary Files

Supplementary File 1
