# Supplementary material for: Identification of a Candidate Rad1 Subunit for the Kinetoplastid 9-1-1 (Rad9-Hus1-Rad1) Complex
Source: Biology (Basel). 2014 Dec 19;3(4):922–7. doi: 10.3390/biology3040922 (PMC4280517; doi:10.3390/biology3040922)
Supplement: Supplementary File 1 [file biology-03-00922-s001.pdf]

Supplementary Material

Table S1. UniProtKB accession numbers for 9-1-1 complex proteins and PCNA from four kinetoplastid organisms, humans and fission yeast.

	**9-1-1 Complex**			**PCNA**
	**Rad9**	**Hus1**	**Rad1**	
T. brucei	Q38EW7	Q581V9	Q4GZB2	Q38F34
T. cruzi	Q4CVH1	Q4DCF3	Q4DWP1	Q4CQ46
L. major	Q4QF86	Q4QBE5	Q4QD01	Q4QF35
A. deanei	S9TXI3	S9U8E7	S9WEJ7	S9X598
H. sapiens	Q99638	O60921	O60671	P12004
S. pombe	P26306	P78955	P22193	Q03392
